# Identification and molecular characterisation of a fibrinogen binding protein from *Streptococcus iniae*.

**DOI:** 10.1186/1471-2180-8-67

**Published:** 2008-04-23

**Authors:** Justice CF Baiano, Reiny A Tumbol, Aarti Umapathy, Andrew C Barnes

**Affiliations:** 1 Aquatic Animal Health Laboratory, Centre for Marine Studies, The University of Queensland, St. Lucia, 4072, Australia; 2 School of Integrative Biology, The University of Queensland, St. Lucia, 4072, Australia

## Abstract

**Background:**

Binding of serum components by surface M-related proteins, encoded by the *emm *genes, in streptococci constitutes a major virulence factor in this important group of organisms. The present study demonstrates fibrinogen binding by *S. iniae*, a Lancefield non-typeable pathogen causing devastating fish losses in the aquaculture industry and an opportunistic pathogen of humans, and identifies the proteins involved and their encoding genes.

**Results:**

Fibrinogen binding by *S. iniae *significantly reduced respiratory burst activity of barramundi peritoneal macrophages in primary cultures compared to BSA-treated or untreated controls, indicating a potentially important role for fibrinogen binding cell-surface proteins in avoiding phagocytic attack in fish. We describe a novel *emm*-like gene, *simA*, encoding a 57 kDa fibrinogen binding M-like protein in *S. iniae*. These SiM proteins and their corresponding tetrameric structures from some sequevar types (~230 kDa) bound fibrinogen in Western blots. *simA *was most closely related (32% identity) to the *demA *gene of *S. dysgalactiae*. Genome walking and sequencing determined the genetic organization of the *simA *region had similarities to the *mgrC *regulon in GCS and to *S. uberis*. Moreover, a putative multigene regulator, *mgx *was orientated in the opposite direction to the *simA *gene in common with *S. uberis*, but contrary to findings in GAS and GCS. In GAS, diversity among *emm*-genes and consequent diversity of their M-related proteins results in substantial antigenic variation. However, an extensive survey of *S. iniae *isolates from diverse geographic regions and hosts revealed only three variants of the gene, with one sequevar accounting for all but two of the 50 isolates analysed.

**Conclusion:**

These proteins play a role in avoiding oxidative attack by phagocytic cells during infection of fish by *S. iniae*, but genetic diversity amongst these key surface proteins has not yet arisen. This lack of diversity coupled with a functional role in macrophage resistance suggests that these proteins may constitute important targets for future vaccines against *S. iniae *in fish.

## Background

One of the most devastating diseases to warm water finfish aquaculture is caused by *Streptococcus iniae*. First isolated from a captive Amazon River dolphin, *Inia geoffrensis*, in 1976, *S. iniae *has caused outbreaks of disease in Israel, Australia, Japan, and the United States [1]. The economic losses attributed to *S. iniae *are estimated to be up to $US10 million annually in the US and over $US100 million globally [2]. Infection in farmed fish usually presents as small red lesions on the skin followed by exophthalmia, and it is found systemically in the blood, kidneys and brain [1]. It is also a known zoonotic pathogen causing a handful of infections to date mainly in elderly people. *S. iniae *is a beta-haemolytic species that does not belong to any Lancefield group[3,4].

It is well established that other streptococcal species such as *S. pyogenes *(GAS), *S. equi *subsp. *zooepidemicus *(GCS), and *S. dysgalactiae *subsp. *equisimilis *(GCS/GGS) possess M or M-like proteins [5-8]. These proteins are located on the surface of the bacterial cell and are considered in GAS to be the dominant virulence factor allowing evasion of phagocytosis and host cell attachment [9]. M proteins are coiled-coil proteins [9,10] characterised by having a signal sequence followed by a hypervariable region, a central region containing a number of repeats, and a cell wall anchor motif [10].

The genes upstream and downstream of the *emm *gene vary from group to group and within species. In GAS, the multigene regulator gene, *mga*, is upstream of the *emm *gene and a C5a peptidase gene is downstream. However, some strains of GAS can also have an *emm*-related protein gene and an *enn *gene [6]. Variations in the *emm *gene region of the genome in GCS and GGS have also been noted where the multigene regulator, *mgc*, is upstream of the *emm *gene, but no other *emm*-like genes are found [6]. Instead, the *cpdB *gene is either present or absent between the *emm *gene and the *rel *gene.

One of the functions of the M protein is the binding of fibrinogen [9,11-13]. In strains of *S. dysgalactiae*, the M protein has also been reported to bind other blood components such as serum albumin, IgG, IgA, and plasminogen [13]. Ability to bind fibrinogen is recognised as a major factor in resistance to phagocytosis [9,11,14]

In this paper, we describe for the first time the *emm*-like genes, *simA *and *simB*, from a Lancefield-untypeable *Streptococcus *species – *Streptococcus iniae*. In other streptococci, M-like proteins are highly diverse contributing to substantial antigenic diversity, however in a survey of 50 isolates from disparate hosts and geographical origin, only 3 variants of the *sim *gene were identified in *S. iniae*. The genetic organization in the vicinity of the *sim *genes is similar to other streptococci, including a multigene regulator upstream with a putative toxic anion resistance gene downstream. The SiM proteins encoded by the *simA *and *simB *genes bind fibrinogen and we demonstrate that fibrinogen binding by *S. iniae *reduces respiratory burst activity in barramundi peritoneal macrophages indicating a likely role in phagocytic evasion and virulence in teleost fish.

## Results

### Fibrinogen binding by *S. iniae*

**Figure 1:**
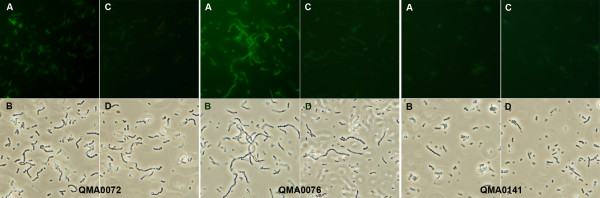
**Fluorescence and phase contrast micrographs of *S. iniae *showing binding of biotinylated fibrinogen**. Strains QMA0072, QMA0076 and QMA00141 were incubated with biotinylated human fibrinogen, extensively washed and subsequently labelled with FITC-conjugated streptavidin. In each case panel A) indicates bacteria incubated with biotinylated fibrinogen, extensively washed and then stained with streptavidin-FITC under fluorescence, whilst B) is the same field photographed under phase contrast. Panel C) indicates control bacteria, incubated in PBS, extensively washed and stained with streptavidin-FITC to detect any non-specific binding of streptavidin, or autofluorescence whilst D) is the same field observed under phase contrast. For fluorescence microscopy, all fields were exposed manually at ISO800 for 1.3 seconds whilst phase contrast fields were captured using automatic exposure.

**Figure 2:**
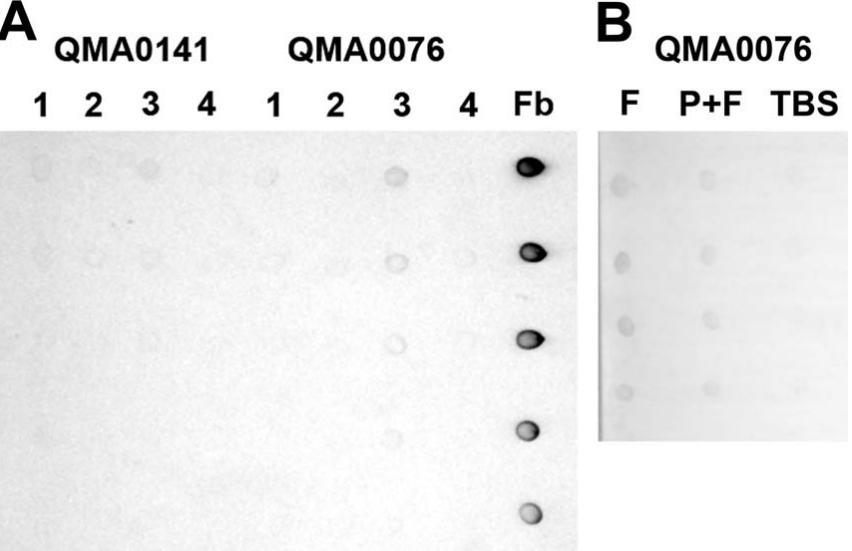
**Dot blots showing binding of biotinylated fibrinogen by *S. iniae***. Strains QMA0076 and QMA0141 were incubated with biotinylated fibrinogen, washed and transferred to PVDF, then membranes were probed with streptavidin-alkaline phosphatase conjugate and developed with NBT/BCIP. Panel A) Lanes 1 and 2 comprised 5 μl cells grown in vegetable peptone, whilst lanes 2 and 3 contained 5 μl cells grown in barramundi serum. Cells were harvested and washed in TBS before being incubated with biotinylated human fibrinogen (Lanes 1 and 3) or TBS (lanes 2 and 4) for 20 min, prior to extensive washing and transfer onto PVDF membrane for subsequent detection. Fb indicates serial two-fold dilutions of biotinylated fibrinogen commencing at 2.5 μg/mL. Panel B) shows serial 2 × dilutions of 5 μl cells diluted in TBS. Lane F, cells incubated first with TBS then with biotinylated fibrinogen before transfer to the membrane. Lane P+F, cells incubated with barramundi plasma followed by biotinylated fibrinogen, Lane TBS, cells incubated twice in TBS.

Wild-type *Streptococcus iniae *QMA0076 and QMA0072 were capable of binding biotin-labelled human fibrinogen *in vitro *as evidenced by fluorescence microscopy of cells counter-labelled with avidin conjugated FITC (Figure 1). This was corroborated for strain QMA0076 by an independent dot-blot assay in which whole cells incubated with biotin-labelled fibrinogen and extensively washed were spotted onto PVDF membrane that was subsequently probed with alkaline phosphatase-conjugated avidin (Figure 2a). QMA0141 did not bind significant quantities of labelled fibrinogen by either method (Figures 1 and 2a). Interestingly, fibrinogen binding was only observed when cells were cultured on blood agar or grown in barramundi (*Lates calcarifer*, Bloch) serum, and not when grown in vegetable peptone broth (Figure 2a). Pre-incubating with fresh barramundi plasma prior to incubation with biotinylated fibrinogen reduced fibrinogen binding ability, but only marginally (Figure 2b)

### Effect of Fibrinogen binding on activation of barramundi peritoneal leucocytes

**Figure 3:**
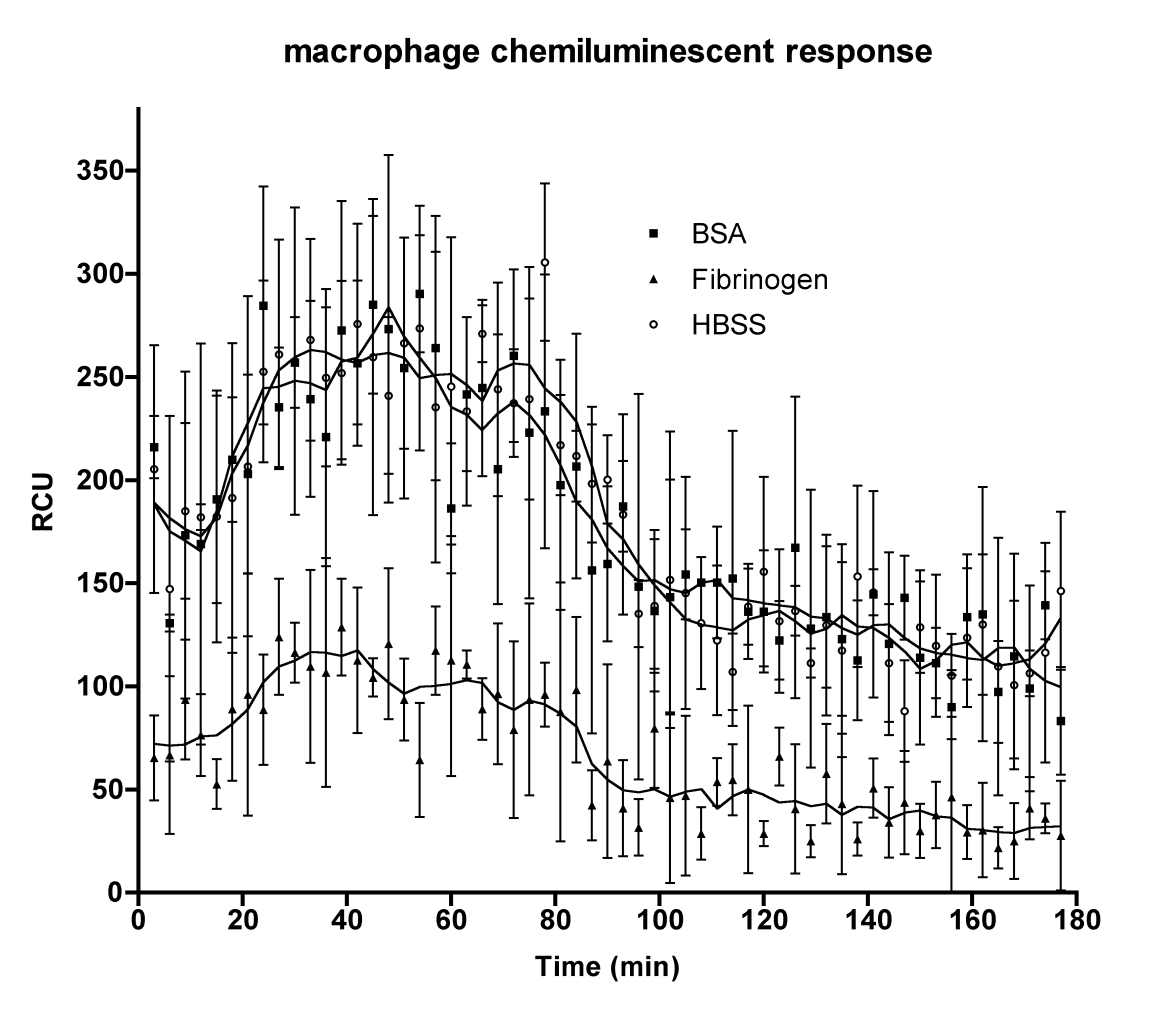
**Effect of fibrinogen binding on activation of barramundi macrophages by *S. iniae***. *S. iniae *isolate QMA0072 harvested from blood agar was incubated with fibrinogen, BSA or HBSS and the respiratory burst was measured by luminol-enhanced chemiluminescence.

To investigate the role of fibrinogen binding by *S. iniae*, the effect of pre-incubating *S. iniae *QMA0072 with fibrinogen on subsequent phagocytosis and respiratory burst in barramundi peritoneal leucocytes was investigated. Fibrinogen binding by QMA0072 significantly decreased subsequent phagocytosis-induced respiratory burst in barramundi peritoneal leucocytes when compared to controls pre-incubated with the same concentration of BSA or in PBS alone (Figure. 3).

### *S. iniae emm*-like (*sim*) genes

**Figure 4:**
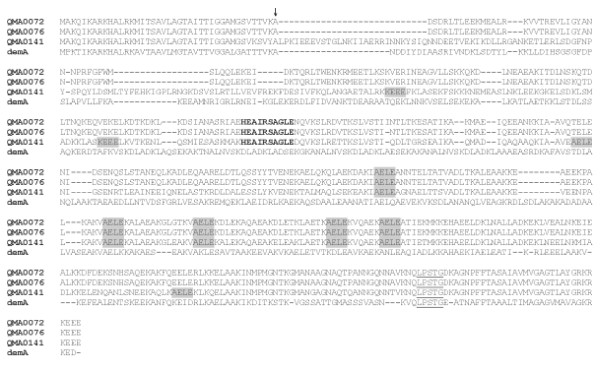
**Amino acid sequence alignment of *S. iniae *M proteins with *S. dysgalactiae demA *gene product (CAB65411)**. Putative signal cleavage site between amino acids 41 and 42 is indicated with an arrow. Repeat regions are highlighted. The putative hinge between the two coils is in bold type and the conserved LPXTG membrane anchor near the C-terminal is underlined.

In order to find genes for putative fibrinogen binding proteins in *S. iniae *a homology cloning strategy was employed using a number of previously published and novel primers designed based on streptococcal *emm *and *emm*-like genes (Table 1). The previously published ALL M primer pair (Table 1) enabled sequencing of the full length *sim *(*Streptococcus iniae emm*-like) gene. Primers for genome walking were designed to determine the true sequence of the gene for the extreme 5' and 3' end nucleotides since the ALL M primer pair introduced artefactual nucleotides (spacer region and encoding amino acids MA at the 5' end and KRKEEN at the 3' end) to the initially deduced sequence. The full length of the *simA *gene was determined to be 1566 bp encoding a 521 amino acid protein in most strains (Figure 4), however, the protein for isolate QMA0072 had one amino acid insertion, and the *sim *gene for isolate QMA0141 (encoded by *simB*) was 579 amino acids (Table 3). The respective molecular masses of the proteins (with signal peptides) for isolates QMA0072, QMA0076, and QMA0141 were 57,589, 57,467, and 63,667 Da respectively. The mature proteins have molecular masses of 53,416, 53,303, and 59,446 Da respectively. There were changes to the amino acid sequence at the 3' end of the nucleotide sequence encoding for KRKEEE. Sequencing of the *simA *gene of *S. iniae *followed by translation from strain QMA0076 revealed that it was distinct from the other known M and M-like protein types from *S. pyogenes, S. suis, S. equi *subsp. *equi*, *S. dysgalactiae*, and *S. uberis*. Sequence alignment of the different types of *sim *gene products from *S. iniae *with their closest matching counterpart, the *demA *gene product from *S. dysgalactiae*, are presented in Figure 4.

**Table 1:** Primers used in this study.

**Primer**	**Sequence (5'-3')**	**Reference**
ALL MF	GGGGGGGGATCCATAAGGAGCATAAAAATGGCT	Podbielski *et al*., 1991
ALL MR	GGGGGGGAATTCAGCTTAGTTTTCTTCTTTGCG	Podbielski *et al*., 1991
SP6	GCTATTTAGGTGACACTATAGAAT	Invitrogen
T7	GTAATACGACTCACTATAGGG	Invitrogen
SIM F	AATTAATGAAGCTGGAGTGCTCT	This study
SIM WF	GGGAGGCTTCGCTGACATTTATTTCC	This study
SIM W3F	TACGGCCGTAAACGCAAAGAGGAAGAA	This study
SIM R	GCTTCCACAAGTTTTTCTTTGTCA	This study
SIM 2R	GTAGATAGGCTTTGATTTTCACTA	This study
SIM 2F	GATACGCTTCAAAGTTCTTACTAT	This study
SIM 3F	GCTGCTAAGATCAACATGCC	This study
SIM WR	ACCTATAATCAGGTTCTAAATTCGTGGC	This study
SIM W2R	GGAAGATGTGTCCATTGTTTGATGAGATG	This study
PRE SIM	TTGTTGGGTGGAAAAAAGATC	This study
POST SIM	AAACTCAGGGACCAAAAAATTG	This study
SIM3F RC	GGCATGTTGATCTTAGCAGC	This study
M141 F	CAAAATGATCACATCAGC	This study
ESIM 20F	CACCATGGCTAAACAAATCAAAGC	This study
ESIM 1542R	TTCTTCCTCTTTGCGTTTACGG	This study

### Organisation of genes in the vicinity of the *sim *genes of *S. iniae*

**Figure 5:**
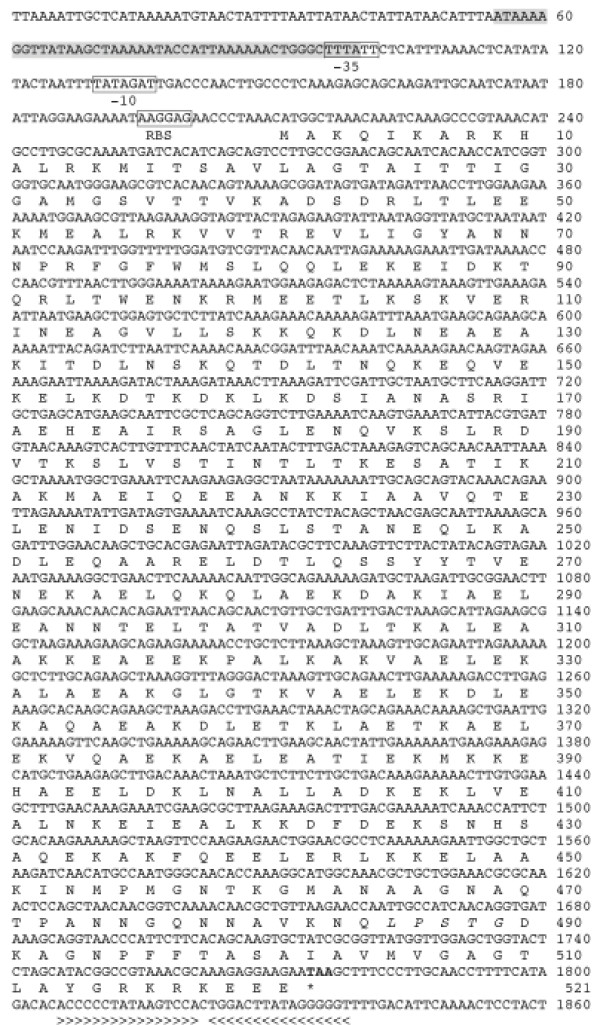
**Nucleotide and amino acid sequences of *S. iniae simA *gene and SiM protein**. Putative Mgx protein binding site is shaded, putative promoter sequences (-10 and -35 boxes and ribosome binding site – RBS – sequence) are boxed, inverted repeats are highlighted by wedges, the membrane anchor is italicised, the stop codon is bolded and indicated by an asterisk.

Genome walking allowed the determination of the genes immediately surrounding the *simA *gene and their relative translational directions. A probable ribosomal binding site with sequence AGGAG was found 11 bases upstream of the start of transcription of the *simA *gene (Figure 5). A putative regulatory protein binding site 45 nucleotides in length was found with a recognition site very similar to that found in the promoter regions of the *emm *and *scpA *genes in GAS [15] (Figure 5). Inverted repeats that occur between the ends of the *simA *gene and *telX *are likely to be a putative rho-independent transcriptional terminator (Figure 5).

**Figure 6:**
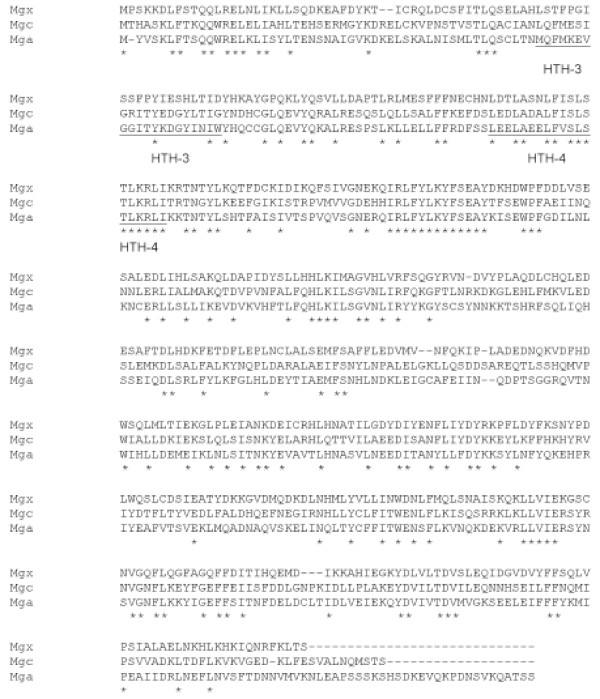
**Amino acid alignment of *S. iniae *Mgx protein with Mgc from *S. dysgalactiae *subsp. *equisimilis *and Mga from *S. pyogenes***. Identical residues are indicated with asterisks. Helix-turn-helix regions are underlined (after Vahling and McIver, 2006).

At the 5' end of the *simA *gene (261 nt upstream) is an ORF of 1488 nt encoding a 495 amino acid protein that shares 39% identity to sequences of Mgc from *S. dysgalactiae *subsp. *equisimilis *(GCS) and 34% identity to Mga for *S. pyogenes *(GAS) (Figure 6). Due to the level of homology, this gene has been designated *mgx *(multigene regulator of Lancefield group unknown streptococci) and the encoded protein is therefore Mgx. The *mgx *gene is orientated in the opposite direction to the *simA *gene. This region of the genome is therefore also named *mgrX *(multigene regulon-like genomic segment in Lancefield group unknown streptococci).

**Figure 7:**
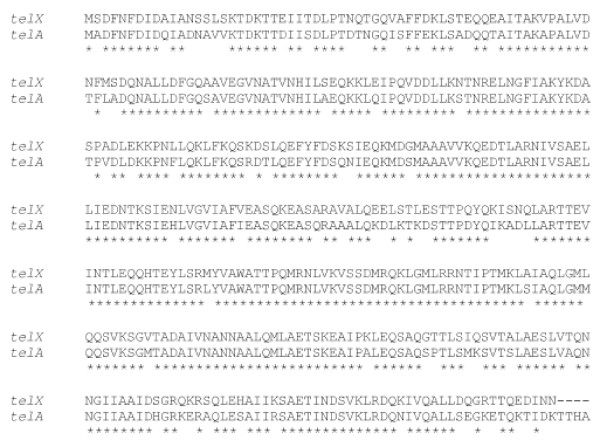
**Amino acid alignment of tellurite resistance gene, *telX*, from *S. iniae *with *S. pyogenes *MGAS8232 *telA***. The proteins share 81% identity and 93% similarity. Asterisks indicate identical residues.

At the 3' end of the *simA *gene, (110 nt downstream) is a gene, orientated in the opposite direction, with high identity (81%; similarity of 93% at the amino acid level) to the tellurite resistance/toxic anion resistance gene *telA *from *S. pyogenes *MGAS8232 (Figure 7). This gene of 1251 nt encodes a protein of 416 amino acids and has been designated as *telX*. Another 20 nucleotides upstream from *telX *is an ORF of 885 nt encoding a 294 amino acid protein which is most closely related to hypothetical gene *Spy_0159 *from *S. pyogenes *M1 GAS with 40% identity and 63% similarity (Figure 7). This gene in *S. iniae *has been called *sihA *(*S. iniae *hypothetical gene A).

A further 110 nucleotides upstream from the start of *sihA *is a probable start for a *purA*-like gene encoding adenylosuccinate synthase (EC 6.3.4.4), although an alternative start codon 305 nt upstream would encode a protein with greater similarity to that from *S. pyogenes *rather than *S. suis*.

**Figure 8:**
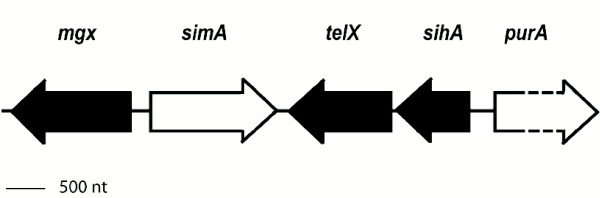
Genetic organization of *the mgrX *segment in *S. iniae *QMA0076.

It was found that the genes *mgx*, *simA*, and *telX *were similar to genes found in other streptococcal species. However, unlike in GAS, GCS and GGS, and as only found thus far in *S. uberis *[16], the *mgx *gene is orientated in the opposite direction relative to the *simA *gene (Figure 8).

### Diversity of *sim *genes in *S. iniae*

In the Group A Streptococci (GAS), diversity of the M proteins accounts for significant antigenic variations and constitutes a major obstacle to vaccine development. By using the PRE SIM and POST SIM primer pair, an expected PCR product of 2048 bp was produced when tested on an additional 49 isolates (Table 2) with only two exceptions. Isolate QMA0072, which had an insertion in the *simA *gene, produced a PCR product of 2051 bp and QMA0141 produced a larger product of 2180 bp. The nucleotide and amino acid sequences for isolates QMA0076 and QMA0072 were identical except for the insertion of three nucleotides (1 aa residue) (Figure 4). The gene sequence for isolate QMA0141 was most divergent having 100% amino acid residue identity for the first 35 residues and 100% similarity for the first 41 residues. This also confirms the theoretical position of where the signal sequence is cleaved to produce the mature SiM protein (Figure 4). This gene was approximately 10% larger than the *simA *gene and was designated *simB*. The nucleotide sequence identity of the *simA *gene in all isolates tested was 100%. The length of the intergenic spacer regions upstream and downstream of the *simA *gene in all isolates was the same size, except for isolate QMA0141 (*simB*), which had a larger sequence downstream. Variations in the upstream intergenic spacer sequence were limited. Thus, the same genetic organization was present in all of the isolates tested.

**Table 2:** Strains used in this study.

**Strain**	**Source**	**Geographic Origin**
QMA0070	*Lates calcarifer*	Townsville, Queensland, Australia
QMA0071	*Lates calcarifer*	Cairns, Queensland, Australia
QMA0072	*Lates calcarifer*	Townsville, Queensland, Australia
QMA0076	*Lates calcarifer*	Townsville, Queensland, Australia
QMA0077	*Lates calcarifer*	Townsville, Queensland, Australia
QMA0078	*Lates calcarifer*	Cairns, Queensland, Australia
QMA0079	*Lates calcarifer*	Cairns, Queensland, Australia
QMA0080	*Lates calcarifer*	Lake Argyle, Western Australia, Australia
QMA0081	*Lates calcarifer*	Lake Argyle, Western Australia, Australia
QMA0082	*Lates calcarifer*	Lake Argyle, Western Australia, Australia
QMA0083	*Lates calcarifer*	Lake Argyle, Western Australia, Australia
QMA0084	*Pteropus conspicillatus*	Western Australia, Australia
QMA0087	*Lates calcarifer*	Lake Argyle, Western Australia, Australia
QMA0109	*Lates calcarifer*	Northern Territory, Australia
QMA0112	*Lates calcarifer*	Northern Territory, Australia
QMA0123	*Lates calcarifer*	Northern Territory, Australia
QMA0125	*Lates calcarifer*	Northern Territory, Australia
QMA0126	*Lates calcarifer*	Northern Territory, Australia
QMA0130	*Homo sapiens*	Canada
QMA0131	*Homo sapiens*	Canada
QMA0133	*Homo sapiens*	USA
QMA0134	*Homo sapiens*	USA
QMA0135	*Homo sapiens*	USA
QMA0137	*Homo sapiens*	USA
QMA0138	*Homo sapiens*	USA
QMA0139	Unknown fish	Canada
QMA0140	*Inia geoffrensis*	USA
QMA0141	*Inia geoffrensis*	USA
QMA0143	*Lates calcarifer*	Northern Territory, Australia
QMA0145	*Lates calcarifer*	Northern Territory, Australia
QMA0150	*Lates calcarifer*	Northern Territory, Australia
QMA0151	*Lates calcarifer*	Northern Territory, Australia
QMA0152	*Lates calcarifer*	Northern Territory, Australia
QMA0156	*Lates calcarifer*	New South Wales, Australia
QMA0158	*Lates calcarifer*	South Australia, Australia
QMA0159	*Lates calcarifer*	South Australia, Australia
QMA0160	*Lates calcarifer*	South Australia, Australia
QMA0166	*Lates calcarifer*	Bowen, Queensland, Australia
QMA0167	*Lates calcarifer*	Bowen, Queensland, Australia
QMA0169	*Lates calcarifer*	Kelso, New South Wales, Australia
QMA0170	*Lates calcarifer*	Bowen, Queensland, Australia
QMA0171	*Lates calcarifer*	Bowen, Queensland, Australia
QMA0173	*Lates calcarifer*	South Australia, Australia
QMA0177	*Lates calcarifer*	Northern Territory, Australia
QMA0182	*Lates calcarifer*	Northern Territory, Australia
QMA0184	*Lates calcarifer*	Northern Territory, Australia
CII-5b-88	*Channa striata*	Bangkok, Thailand
KFP173	*Oncorhynchus mykiss*	Upper Galilee, Israel
KFP404	*Oncorhynchus mykiss*	Upper Galilee, Israel
21–96(2)	*Oncorhynchus mykiss*	Réunion Island

**Table 3:** *S. iniae sim *gene lengths and M protein identity.

**Strain**	**Length (nt, aa)**	**BLAST-P result**	**Identity (%)**^a^	**Similarity (%)**^b^	**Gene**	***sim *sequevar**
QMA0072	1569, 522	*S. dysgalactiae demA *gene	32	49	*simA*	A2
QMA0076	1566, 521	*S. dysgalactiae demA *gene	32	50	*simA*	A1
QMA0141	1740, 579	*S. dysgalactiae *subsp. *dysgalactiae *M-like protein	31	48	*simB*	B1

^a,b ^– % according to BLASTP analysis.

### *mgx *sequence analysis

**Figure 9:**
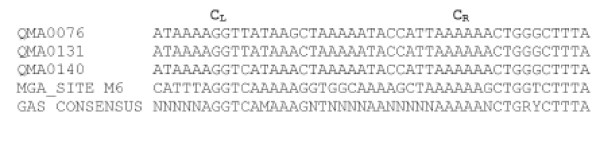
**Alignment of putative Mgx binding sites upstream of *simA *genes**. Alignment shows sequences from different isolates including Mga binding site and GAS consensus sequence (after McIver *et al*., 1995).

The *mgx *gene encodes the protein Mgx which contains helix-turn-helix structures (Figure 6). Sequence alignment with other multigene regulators from *S. pyogenes *and *S. dysgalactiae *subsp. *dysgalactiae *showed that there was a high level of conservation in the HTH-4 helix-turn-helix domain from residues 106–125 with these Mgx homologues. This is a common feature of all multigene regulator proteins found in GAS and GCS/GGS and from some unfinished genome projects and is necessary for transcriptional activation of virulence factors [17]. The multigene regulator in *S. iniae *was more divergent as a whole, than those of other streptococcal groups, especially in the other known helix-turn-helix region (HTH-3) (Figure 6). Putative Mgx protein binding motifs were found in the spacer regions upstream of the *simA *or *simB *genes in all isolates sequenced (Figure 9). Variations in Mgx binding sites between isolates may be due to differences in the Mgx protein in different isolates. Like the binding sites in other species, it is 45 bp long and overlaps the -35 promoter box [15]. The region on the right hand side of the consensus sequence (C_R_) is more conserved than that from the left (C_L_) [15] (Figure 9).

### SiM protein sequence analysis

The N-terminal of the major SiM protein type from isolate QMA0076 has a probable signal sequence cleavage site between amino acids 41 and 42 resulting in a mature protein of 480 amino acids with a molecular mass of ~53 kDa (Figure 4). For isolate QMA0141, the signal peptide cleavage point is also between residues 41 and 42 resulting in a mature protein of 538 residues with a molecular mass of ~59 kDa (Figure 4). In the C terminal end of all of the SiM proteins is the conserved Gram positive cell wall anchor motif LPSTG [18]. The N-terminal region of the SiM protein types had very similar signal peptides, but the first residues of the mature protein differed significantly while there was a high level of conservation at the C terminal ends of the proteins. This indicates that selective pressures have been exerted upon the exposed N terminal parts of the protein.

Analysis by the Garnier algorithm [19] for isolate QMA0076 predicted that 77.2% of the protein was alpha-helical. For isolates QMA0072 and QMA0141, the predicted values were 77.1% and 77.4% respectively for their SiM proteins.

Analysis by the COIL program [20,21] with a window length of 28 predicted that the protein from isolate QMA0076 (with signal sequence) has two coiled-coil segments from residues 79–174 and 181–454 with a probability of 1.00 for each segment. This is a similar result to that of Vasi *et al. *(2000)[18] who identified M-like proteins from *S. dysgalactiae*. By weighting the first and fourth residues in the heptad repeats a probability of 1.00 was gained for residues 91 to 449. Similarly, for isolate QMA0072 two coiled-coil segments from residues 79–174 and 196–455 with a probability of 1.00 for each segment were predicted. Weighting the first and fourth residues in the heptad repeats gave a prediction with a probability of 1.00 for two segments from residues 91–186 and 213–450.

The SiM protein from isolate QMA0141 showed a probability of 1.00 that there was a coiled-coil structure from residues 154–512 with a weighted heptad, and coiled-coils at residues 151–231 and 238–512 when an unweighted heptad was used.

The SiM proteins also share repeat elements that are a feature of M proteins. QMA0072 and QMA0076 contained five repeat elements of the residue sequence AELE. In contrast, the SiM protein from isolate QMA0141 contained five pairs of repeat elements represented by KEEE, AELE, AKVAELEKAL, ELEA, and KAELEK. Large peptide sequence motifs conserved across all SiM proteins are HEAIRSAGLE between the coils, the signal peptide, and the C terminal end which has few variations over the last ~255 residues of the protein.

### PAGE and Western blot analysis of expressed SiM proteins

**Figure 10:**
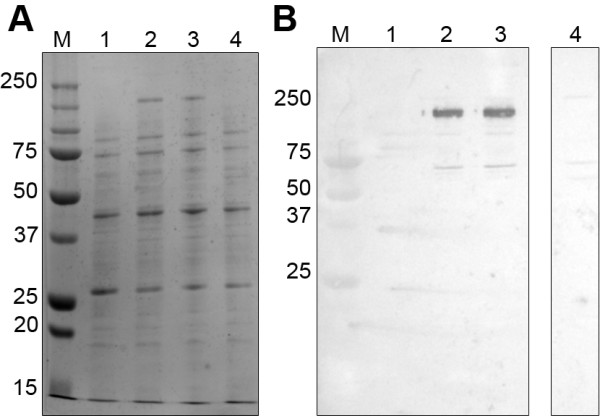
**Western blot of SDS-PAGE separated lysates of *E. coli *expressing recombinant M proteins**. Lane M – molecular weight marker, lane 1 – control lysate, lane 2 – QMA0072, lane 3 – QMA0076, lane 4 – QMA0141. A) Transferred lysates stained with Coomassie Brilliant Blue R250. B) Detection of recombinant SiM proteins in *E. coli *lysates with biotinylated fibrinogen.

Analysis by SDS-PAGE of non-reduced expressed proteins (Fig. 10A) showed that there were bands present which were different to that contained in the control *E. coli *lysate. Prominent protein bands were present at ~230 kDa from isolates QMA072 and QMA076; but not for isolate QMA0141.

Western blotting was used to detect the fibrinogen binding proteins from these lysates. Binding of biotinylated fibrinogen followed by detection with alkaline phosphatase conjugated streptavidin in Western blots of SDS-PAGE separated lysates from *E. coli *expressing recombinant SiM proteins showed that the SiM proteins from isolates QMA0072 and QMA0076 appeared as monomeric of ~57 kDa as expected and also as tetramers of ~230 kDa. A band that weakly bound fibrinogen was also observed that appears to be a dimer at ~115 kDa. The SiM protein from isolate QMA0141 appeared as bands at ~64 kDa and ~255 kDa and does not appear to bind fibrinogen as strongly as that from isolates QMA0072 and QMA0076 (Fig. 10B). The formation of tetramers has been observed in SiM proteins from other species [12].

## Discussion

Evasion of phagocytosis is critical in the pathogenic strategies of many of the streptococci [9]. In *S. iniae*, capsule has been identified as a major virulence factor [22,23] contributing to resistance to serum killing [24] and phagocytic evasion [25]. In other streptococci, binding of serum components by surface associated M-like proteins constitutes a major antiphagocytic mechanism. In the present study, we have demonstrated fibrinogen binding by whole *S. iniae *cells *in vitro*. Biotinylated fibrinogen that bound to the cell surface could be detected by fluorescence microscopy using fluorescein conjugated streptavidin. Because the fluorophore faded quickly under fluorescence microscopy an alternative dot-blot method was developed using an alkaline phosphatase conjugate. This enabled semi-quantitative evaluation of fibrinogen binding and indicated that strain QMA0141 bound less fibrinogen than QMA0076. This was supported by Western blots using the recombinantly expressed proteins from these strains and is reflected in the differing *sim *gene sequences of these isolates. As an indication of the potential role of fibrinogen binding by *S. iniae*, *S. iniae *cells incubated with fibrinogen prior to exposure to barramundi macrophages significantly reduced the subsequent respiratory burst of the macrophages, indicative of reduced phagocytosis.

Interestingly, fibrinogen binding was not detected when strains were grown overnight in vegetable peptone broth, and was only observed following growth on blood agar or in heat inactivated barramundi serum. This suggests that these proteins may be the same proteins previously reported to bind trout immunoglobulins, or are under the same regulatory control [26]. In the present study, we found a putative multigene regulator, encoded by *mgx*, upstream of the *simA *or *simB *gene. The *sim *genes from *S. iniae *are, in common with *emm *and *emm*-like genes, most likely to be controlled by this multigene regulator; a 45 bp putative DNA binding site for the Mgx protein lies between bases 184 and 140 upstream of the *sim *genes [27,28] and overlaps the -35 RNA polymerase promoter box as has been determined for GAS [15]. Further studies are ongoing to determine if, as in GAS with Mga, the Mgx protein binds to this promoter region and activates transcription of *sim *and also to identify if Mgx is involved in its own regulation [29]. Additionally, the environmental conditions that govern regulation of these genes are being explored in order to optimise expression in culture.

The SiM proteins are quite different from previously reported M proteins in other streptococci, with the closest relative being the *S. dysgalactiae dem *gene at only 32% identity. In terms of the genetic organization of the *MgrX *segment, some similarities were found not only with GAS and GCS but also with the unusual *S. uberis*. In *S. iniae *the *mgx *gene was organised in reverse orientation to the *sim *genes. This reversal has only been found to date in GES/GBS *S. uberis *where the genetic organization of the multigene regulator and the lactoferrin binding protein are in opposite directions [16]. This differs from GAS, GCS and GGS where the orientation of the multigene regulatory and *emm, emm-*like and *dem *genes is in the same direction.

Similarities with GAS were found from the many genome sequencing projects from GAS serotypes M1, M3, M5, M6, M18 and M28 [27,28,30-34]. In all of these genomes, the genes for *telA*, *Spy_0159 *(and other names given to this hypothetical gene), and *purA *are located side by side and in the same relative transcriptional directions as *telX*, *sihA*, and *purA *in *S. iniae*. The genes upstream of *telA *in GAS may be involved with energy production and conversion, however, downstream of the *purA *gene is a putative lipoprotein ABC transporter gene and a streptolysin O precursor gene, which may indicate a role in virulence for these genes. The roles of these homologous genes in *S. iniae *may also be similar, especially when their proximity to *mgx *is considered, and the potential for other virulence factors that may be located nearby.

As evidenced by Western blotting of recombinant SiM proteins expressed in *E. coli*, the SiM protein from isolate QMA0076 appeared to bind the most fibrinogen, with that from isolate QMA0141 binding very little fibrinogen following transfer to the PVDF membrane. The one amino acid residue insertion after the intercoil region (in the second coiled region) of isolate QMA0072 did not appear to significantly disrupt the fibrinogen binding ability of the coils. It is interesting to note that all SiM protein sequences, regardless of size or divergence, contain the peptide sequence HEAIRSAGLE that connects the two coils. Conservation of this peptide indicates that it is likely to have a critical structural role for the binding of blood components such as fibrinogen and immunoglobulins [13] and needs to be investigated further. The signal peptide and C terminal ends were well conserved in all SiM types indicating that very little selective pressure has been exerted on these regions.

The ability to form tetrameric conglomerations may also be due to the coiled regions interacting with one another since the apparent molecular mass of ~230 kDa has also been observed with a fibrinogen binding protein from *S. equi *subsp. *equi *[12]. It is interesting to note that the ability of the SiM protein from isolate QMA0141, which had a much larger molecular weight, did not bind fibrinogen as efficiently as the major type. Given that other M and M-like proteins are able to bind other blood proteins, the ability of these proteins to bind them also cannot be discounted. Indeed, a previous study reported binding of immunoglobulin from normal trout serum by *S. iniae *proteins of a similar molecular weight to the tetramers described in this study [26].

The lack of diversity of the *sim *genes present in our strain collection, which represents isolates from disparate geographic location and hosts, is surprising, especially when viewed in the context of *emm *gene sequence diversity found in GAS [35]. Only three gene types were found, with two of these types occurring only once each (Table 3). This may simply be a result of functional conservation, with sequence variation resulting in reduced binding capacity, as indicated in the present study with isolate QMA0141, leading to strains with this gene type to be relatively unsuccessful. An alternative hypothesis may be that there has been little or no selective pressure applied by the infected fish host to drive a change in this protein: perhaps as result of the organization of the fish immunogloblin light chains [36], they appear to be able to recognise a less diverse repertoire of epitopes than their mammalian counterparts [37] and SiM proteins simply may not be immunogenic in fish. This is corroborated by previous work in trout and by observations in our own laboratory that antiserum from whole-cell immunised barramundi do not detect SiM proteins in Western blots (unpublished data). An alternative explanation is that once fish are infected with *S. iniae*, the opportunity to produce antibodies is negated by the short period of time between infection and death. The introduction of vaccination programmes against *S. iniae *into warm water aquaculture may provide the selective pressure required to drive evolution of further diversity in these genes.

In the present study, genes encoding a fibrinogen-binding M-like protein have been cloned, sequenced and expressed as recombinants for the first time from this pathogen of major importance in global aquaculture. It has been shown that members of the M protein family contribute to virulence of *Streptococcus *sp., have the ability to bind blood components other than fibrinogen, and have a role in resistance to phagocytosis [9,11,12,38-41]. The present results support this observation, indicating that binding of fibrinogen by *S. iniae *reduced respiratory burst activity in fish macrophages. Moreover, an earlier study indicated proteins with a similar apparent molecular weight to those reported in this study are capable of binding trout immunoglobulins in reverse orientation (i.e. by the Fc region) [26]. Fibrinogen binding by *S. iniae *was only apparent in cultures grown on blood agar or in barramundi serum, indicating that SiM expression is likely to be environmentally regulated. Our identification of a putative regulatory protein and binding site upstream of the *sim *genes will enable further research into the control of these genes. The M protein is a major virulence factor and vaccine candidate in GAS [42-45] and future work is directed towards investigating the potential of a vaccine against *S. iniae *infection in farmed fish based on these intriguing proteins.

## Experimental Procedures

### *S. iniae *strains and culture conditions

Veterinary laboratory isolates of *S. iniae *taken from infected barramundi (*Lates calcarifer*), along with reference isolates from the Centers for Disease Control, Atlanta, and further global isolates were used in this study (Table 2). Strains in 20% glycerol that were stored at -80°C were grown overnight at 37°C on Columbia agar base containing 5% defibrinated sheep blood. For liquid cultures, *S. iniae *was grown in 10 ml vegetable peptone broth (Oxoid, Basingstoke, UK) in sterile 15 ml centrifuge tubes (Greiner Bio-One, Germany) statically at 37°C.

### Whole cell fibrinogen binding assay

Human fibrinogen (Sigma, Castle Hill, Australia) was labelled using the EZ-Link Sulfo-NHS Biotinylation Kit (Pierce, Rockford, Il.) in accordance with the manufacturers instructions. Unincorporated biotin was removed using Micro-Spin G-25 columns (GE Healthcare Biosciences, North Ryde, Australia). Barramundi blood was collected by caudal venipuncture from 150 g fish using a 1 ml tuberculin syringe fitted with a 23 G needle. For serum, blood was allowed to clot for 24 hours at 4°C to ensure complete removal of fibrinogen before centrifugation at 3000 × *g *for 10 min to remove blood cells. For collection of plasma, 50 μL ice-cold heparin (10 U/mL) was drawn into the syringe prior to blood collection. Blood cells were removed by centrifugation at 3000 × *g *and plasma was either used immediately or stored at -20°C until required.

*S. iniae *cultured overnight on blood agar, in 1 ml heat inactivated barramundi serum or in vegetable peptone broth at 28°C was washed and suspended in phosphate buffered saline (PBS) to an optical density of 2.5 at 600 nm. Aliquots (200 μL) were then incubated in Tris buffered saline (TBS) containing 5 μg/mL biotinylated fibrinogen, or in TBS alone as a control for 20 min at room temperature. Cells were harvested at 6,000 × *g *for 5 min in a microcentrifuge and then washed six times in 1 mL TBS to remove any unbound fibrinogen. For fluorescence detection of bound fibrinogen, washed test and control cells were incubated with streptavidin-conjugated fluorescein isothiocyanate (FITC) diluted to 25 μg/mL in PBS for 30 min. Cells were then washed eight times in PBS before resuspension in 50 μL PBS. Aliquots (10 μL) were then transferred onto washed glass microscope slides and covered with a glass cover slip. These wet mounts were then viewed under phase contrast using and Olympus BX41 epifluorescence microscope. As FITC fades rapidly upon UV exposure a procedure to eliminate exposure prior to capture was optimised as follows. Once a suitable field had been located under phase contrast it was photographed using the attached Olympus DP70 digital video camera. With the shutter between the stage and mercury vapour lamp closed to prevent UV exposure of the labelled cells, the FITC filter was moved into position and the camera setting adjusted manually to ISO800 and a shutter speed of 1.3s. The stage shutter was then opened to expose the cells to UV light and the image was captured simultaneously.

To support results obtained by fluorescence microscopy, a whole cell dot-blot method was used. In this method cells labelled with biotinylated fibrinogen and controls in TBS as described above were serially twofold diluted in TBS in a microtitre plate. A row containing serially diluted (from 2.5 μg/mL) biotinylated fibrinogen was included as a positive control. Aliquots (5 μL) were then transferred to pre-wetted PVDF (Immobilon P, Millipore, UK) using a multichannel pipette and allowed to dry. The membrane was then blocked for 1 hour with 1% vitamin-free casein (Sigma, Castle Hill, Australia) in TBS containing 0.05% Tween 20 (TBST). Following blocking, endogenous phosphatase activity was quenched with 20% acetic acid in distilled water for 5 min. After 3 × 5 min washes in TBST, the membrane was probed with alkaline phosphatase-conjugated streptavidin (0.2 units/mL) in TBST for 30 min. The membrane was then washed (×3) in TBST and then once in TBS before developing in a liquid phosphatase substrate (1-Step NBT/BCIP, Pierce, Rockford Il.) until the colour developed.

### Isolation and culture of peritoneal macrophages from barramundi

Macrophages from casein stimulated peritoneal cavity were harvested, purified and maintained as described previously [46]. Briefly, for stimulation of the peritoneum, barramundi (*Lates calcarifer*) (300 g) were anaesthetisised with Aqui-S (Aquatic Diagnostic Services, Wilston, Australia) in accordance with the manufacturers instructions, and then injected with 1 mL of 12% casein (sterile, in phosphate buffered saline, PBS) into their peritoneal cavity 24 h before collection of macrophages. Prior to the isolation of peritoneal macrophages, fish were euthanised with overdose Aqui-S and then exsanguinated by cutting the ventral aorta. An aliquot (5 mL) L-15 medium containing 2% Foetal Bovine Serum (FBS, Invitrogen, Melbourne, Australia), 1% penicillin/streptomycin (P/S) (Invitrogen, Melbourne, Australia), and 10 U/mL heparin (Sigma, Castle Hill, Australia) was injected aseptically into the peritoneal cavity using a syringe fitted with a 25 G needle. The body cavity was then massaged for 30 s to disperse the medium and the lavage containing leucocytes was withdrawn using 19 G syringe very carefully to prevent bleeding. The suspensions of peritoneal cells were then layered onto a discontinuous (34%/51%) Percoll density gradient and centrifuged at 450 g for 25 min at 4°C. The band lying at the interface was collected and washed twice with L-15 medium containing1% FBS and 1% P/S. Concentration of viable cells was determined by Trypan blue exclusion. The cells were seeded in microtiter plates at the concentration of 10^7 ^cells/mL in L-15 medium with1% FBS and 1% P/S. Cell populations were allowed to adhere for 2 h at 28°C and then washed twice with L-15 medium to remove the unattached cells. The adhered cells were maintained in L-15 with 1% FBS and 1% P/S at 28°C. For the assay, 24 h cultures were used.

### Induction of luminol-amplified chemiluminescence in peritoneal macrophages

*Streptococcus iniae *strain QMA0072 was used for this assay. A suspension of cells taken directly from blood agar cultures (OD_600 _= 1.5) was washed in PBS then pre-incubated in PBS, 5 μg/mL bovine serum albumen (BSA) in PBS, or 5 μg/mL fibrinogen in PBS for 30 minutes at 37°C. The cells were then washed twice with HBSS and resuspended in HBSS.

The respiratory burst activity of the peritoneal macrophages following phagocytosis was determined using protocols described previously [47] with some modifications. Briefly, peritoneal macrophages, cultured for 24 h, were incubated with either fibrinogen-treated, PBS-treated or BSA-treated *S. iniae *at a multiplicity of infection (MOI) of 100 (bacteria/macrophage ratio 100:1). The respiratory burst activity was initiated by the addition of 30 μL of the required bacterial suspension to macrophages in microtitre plates (1 × 10^5 ^cells per well). To each well, 10 μL of 10 mM luminol in 0.2 M borate buffer pH 9.0 and 280 μL HBSS pH 7.4 were also added to get a final volume of 300 μL. The chemiluminescence (CL) emissions of the phagocytes were measured in a luminometer/fluorometer (BMG Fluostar Optima, BMG Labtech, Offenberg, Germany) every 3 minutes for 3 hours at 27°C.

### Recombinant DNA techniques

*S. iniae *genomic DNA was extracted from freshly-grown cells using an enzymatic lysis method [7].

### PCR Amplification of *S. iniae emm*-like (*sim*) genes and cloning

Each 50 μL PCR tube contained 5 μL of 10 × *Tth *plus buffer, 1 μL dNTP's (4 × 2.5 mM of each of dATP, dCTP, dGTP and dTTP; Biotech International Ltd., Australia), 200 ng of ALL MF and ALL MR primers (Table 1), 0.5 U of *Tth *plus DNA polymerase (Biotech International Ltd., Australia), 3 μL of 25 mM magnesium chloride, and the balance made up with sterile Milli-Q water. Thermal cycling parameters in an Eppendorf Mastercycler Gradient EPS (Eppendorf, Hamburg, Germany) were denaturation for 2 mins at 94°C followed by 35 cycles of 50°C for 1 min, 72°C for 2 min, and 94°C for 15 s with a final extension cycle of 50°C for 1 min and 72°C for 10 min. The resultant PCR products were visualised after electrophoresis in a 1% w/v agarose gel containing 0.5 μL of 10 mg/mL ethidium bromide solution in 1× TAE as the electrophoresis buffer.

Desired bands were excised from the gel and extracted from the agarose with the MegaSpin Agarose Gel Extraction Kit (Intron Biotechnology, Korea). Purified PCR products were ligated into PCR4-TOPO by TA cloning using a commercial kit (Invitrogen, Melbourne, Australia). Competent cells (TOP 10; Invitrogen, Melbourne, Australia) used in cloning experiments were cultured on Luria-Bertani Agar (LB Agar; Sigma, Castle Hill, Australia) supplemented with 100 μg/mL ampicillin. X-gal (40 μl of 20 mg/ml) was spread onto LB agar plates for blue-white discrimination. Clones were grown at 37°C overnight. Clones were picked using a sterile disposable loop and plated onto LB agar. These confirmed white clones were also used for screening by direct lysis PCR and for storage.

### Direct lysis PCR of clones and sequencing

The reaction volume was scaled down to 25 μL, with the same reaction conditions as above. Use of plasmid-specific primers SP6 and T7 (Table 1) enabled the *sim *gene inserts to be amplified. Agarose gel electrophoresis allowed determination of clones with inserts, from which plasmids were extracted using a commercial kit in accordance with the manufacturers instructions (Invitrogen, Melbourne, Australia) for sequencing with plasmid primers SP6 and T7 (Table 1). Sequencing of the full length of the *sim *genes was initially facilitated with the primer SIM F (Table 1).

### Genome Walking and *sim *gene diversity

Sequences upstream and downstream of the *sim *gene were obtained by genome walking according to the manufacturer's instructions (Genome Walker Kit, Clontech, Mountain View, Ca.). Gene specific primers used for genome walking upstream of the *sim *gene were SIM WR and SIM W2R (Table 1) and SIM WF and SIM W3F (Table 1). Resultant PCR products were gel-purified and ligated into TOPO vector PCRII (Invitrogen, Melbourne, Australia). Sequencing was carried out with SP6 and T7 primers on recombinant clones. This allowed primers to be designed (PRE SIM and POST SIM; Table 1) for the amplification of the *sim *genes and the surrounding spacers from additional strains (Table 2) to examine *sim *gene diversity. The same cycling parameters were used but with a 65°C annealing temperature and use of a proofreading polymerase enzyme system (Prime STAR DNA polymerase, Takara, Shiga, Japan) to reduce the likelihood of incorporation errors. The PRE SIM primer was located in the multigene regulator gene (*mgx*) and included the first 36 nucleotides of the *mgx *gene in the amplicons and the POST SIM primer included the last 75 nucleotides of the putative tellurite/toxic anion resistance gene (*telX*). These PCR products were directly sequenced with the following primers: PRE SIM, SIM R, SIM 2R, SIM F, SIM 2F, SIM 3F, SIM W2R, and POST SIM (Table 1). Additionally, primers M141 F and SIM3 F RC were used to generate sequence data from isolate QMA0141.

### Expression of SiM proteins

M proteins were cloned for expression using the Champion pET system (pET101/D-TOPO; Invitrogen, Melbourne, Australia) and subsequently expressed in *E. coli *BL21 Star (DE3) One Shot chemically competent cells (Invitrogen, Melbourne, Australia) according to the manufacturer's instructions. Representatives of the different *sim *sequevars recovered were expressed according to the manufacturer's instructions. Representative isolates of the different *sim *genes QMA0072, QMA0076, and QMA0141 had their full proteins expressed.

To confirm fibrinogen binding activity of recombinantly expressed proteins, a Western blotting procedure was used. Lysates of recombinant E. coli described above were separated by SDS-PAGE in resolving gels containing 10% acrylamide under reducing and non-reducing conditions. Resolved IPTG-induced cellular lysates were blotted onto PVDF membranes using a semi dry apparatus (Hoeffer Semi-Phor, GE Healthcare, North Ryde, NSW), the membranes were probed with biotinylated fibrinogen (5 μg/mL) prepared as described above and detected using streptavidin-alkaline phosphatase (Pierce, Rockford, Il.) followed by colour development using 1-Step NBT-BCIP (Pierce, Rockford Il.).

## Nucleotide Sequence Accession Numbers

GenBank accession numbers for sequences from this study are EU287919–EU287923.

## Authors' contributions

JCFB discovered, characterised, and expressed the *sim *genes, discovered the surrounding genes, designed new molecular materials, drafted the manuscript and supervised AU. RAT contributed with macrophage assays and drafted part of the manuscript. AU contributed with PAGE and detection of proteins on western blots. ACB designed the study, conducted the fibrinogen binding assays, supervised RAT and revised the final manuscript. All authors have read and approved the final manuscript.
